# Database on the coverage of the “Bolsa-Família” conditioning cash-transfer program: Brazil, 2005 to 2021

**DOI:** 10.1186/s13104-021-05849-5

**Published:** 2021-11-27

**Authors:** Lais Baroni, Ronaldo Fernandes Santos Alves, Cristiano Siqueira Boccolini, Rebecca Salles, Raquel Gritz, Balthazar Paixão, Patricia de Moraes Mello Boccolini

**Affiliations:** 1 Federal Center for Technological Education of Rio de Janeiro, CEFET/RJ, Rio de Janeiro, Brazil; 2 Oswaldo Cruz Foundation, Fiocruz, Rio de Janeiro, Brazil; 3 Platform of Data Science applied to Health (PCDaS), ICICT, Oswaldo Cruz Foundation, Fiocruz, Rio de Janeiro, Brazil; 4 Petropolis Medical School, UNIFASE, Petrópolis, Brazil

**Keywords:** Child Health, Social programs, Nutrition Programs and Policies, Vaccination Coverage, Health Information Systems, Database

## Abstract

**Objectives:**

The “Bolsa-Família” Program (PBF) is a Brazilian conditional cash-transfer program in which families should comply with health, education, and social assistance conditionalities. The program aims to fight poverty and hunger, promoting nutrition and health services for low-income populations. This paper presents a database on the coverage of monitoring and compliance with the PBF health conditionalities in Brazil from January 2005 to July 2021.

**Data description:**

Database on the PBF conditioning cash-transfer program coverage in Brazil from 2005 to 2021. It comprises information on the number of families benefited, health conditionalities, and the follow-up on vaccination and nutrition of children under seven years old. The cities and semesters are the minimal aggregation units.

## Objective

The “Bolsa-Família” Program (PBF) is a Brazilian direct cash transfer program in which families should comply with conditionalities on health, education, and social assistance [1, 2]. The PBF aims to protect the human rights of vulnerable families, interrupting the intergenerational cycle of poverty. The PBF contemplates families with monthly per capita income up to R$89.00 (U$17.5) or families with monthly per capita income between R$89.01 and R$178.00 (U$17.5 to U$34.9) that have pregnant women or children up to 17 years old [2]. The primary health care workers of Unified Health System (SUS) are responsible for monitoring and recording compliance with the PBF health conditionalities [2, 3].

It is mandatory to monitor children under seven years old, women from 14 to 44 years old, and pregnant women regarding nutritional status, vaccination, and prenatal and postpartum care [4]. All information about the beneficiaries is registered in the PBF in Health System (BFA) of the Ministry of Health [3]. Regular monitoring of these conditionalities contributes to fulfilling constitutional health commitments and national health policies, improving health care and the population’s health and nutrition conditions. Additionally, the monitoring makes it possible to identify which families have difficulty accessing health services. Based on this information, the government can act to improve actions and services aimed at the most vulnerable populations. The PBF contributes to the reduction of mortality in under-5s, especially deaths attributed to poverty, such as malnutrition and diarrhea [5].

We present a dataset that harmonizes, processes, validates, and enriches the data of the BFA System. The dataset is part of the COVAC [6] and MATRECI [7] studies and will be integrated with data on primary health care coverage and preventable infant mortality in Brazil.

## Data description

Coverage data for monitoring and compliance with PBF health conditionalities by municipality, semester, and year were extracted from the BFA System on July 9, 2021. The BFA System data are available on the former BFA platform^1^ (2005/1 to 2018/1) and on the current e-Gestor AB platform^2^ (2018/2 onwards). The data extraction resulted in 33 files (.csv format), covering January 2005 to June 2021. These files were compiled and processed through a routine developed in the free and open-source software R.

This report provides 37 datasets and 7 data files. The 33 original databases (*pbf_health_data_raw.zip*) and two crude databases (*pbf_health_crude.zip*) – formed by the simple combination of the original files from each BFA platform – are in a compressed folder. Processed databases are available in Portuguese (*pbf_health_full_epi_pt.csv*) and English (*pbf_health_full_epi_en.csv*). The Excel file ‘*pbf_health_overview.xlsx*’ includes Portuguese and English versions of the metadata and variable dictionary of the treated databases. The HTML files (pbf_health_dataselfie_*.html) present statistical summaries of each treated database variable. R scripts hold the codes for the data ingestion/curation routine (*script_pbf_health_ingestion.rmd*) and the data analysis routine (*script_pbf_health_analysis.rmd*). The files ‘ *pbf_health_tables.xlsx*’ and ‘ *pbf_health_figures.pdf*’ gather, respectively, the tables and views generated in the data analysis step (Table 1).

**Table 1:** Overview of data files/datasets

Label	Name of data file/dataset	File type (file extension)	Data repository and identifier
Dataset 1	pbf_health_data_raw	zipped (.zip)	Synapse: https://doi.org/10.7303/syn25884313 [8]
Dataset 2	pbf_health_crude	zipped (.zip)	Synapse: https://doi.org/10.7303/syn25884313 [8]
Dataset 3	pbf_health_full_epi_pt	delimited text (.csv)	Synapse: https://doi.org/10.7303/syn25884313 [8]
Dataset 4	pbf_health_full_epi_en	delimited text (.csv)	Synapse: https://doi.org/10.7303/syn25884313 [8]
Data file 1	pbf_health_overview	excel (.xlsx)	Synapse: https://doi.org/10.7303/syn25884313 [8]
Data file 2	pbf_health_dataselfie_pt	HTML (.html)	Synapse: https://doi.org/10.7303/syn25884313 [8]
Data file 3	pbf_health_dataselfie_en	HTML (.html)	Synapse: https://doi.org/10.7303/syn25884313 [8]
Data file 4	script_pbf_health_ingestion	R code (.rmd)	Synapse: https://doi.org/10.7303/syn25884313 [8]
Data file 5	script_pbf_health_analysis	R code (.rmd)	Synapse: https://doi.org/10.7303/syn25884313 [8]
Data file 6	pbf_health_tables	excel (.xlsx)	Synapse: https://doi.org/10.7303/syn25884313 [8]
Data file 7	pbf_health_figures	Portable document (.pdf)	Synapse: https://doi.org/10.7303/syn25884313 [8]

### Data construction

Intake codes, curation, and descriptive data analysis were developed, examined and their results were compared to the information displayed on the e-Gestor AB platform. Data processing included the activities of (i) renaming the variables; (ii) separation of the field referring to the semester and year of competence; (iii) cleaning of numerical values, e.g., excluding special characters and (iv) enrichment of the municipal database with data aggregated by state, geographic region, and Brazil.

Two business rules were applied to the PBF monitoring coverage percentage indicators: (i) the coverage percentage will be equal to 0%, whenever the denominator value is equal to 0; (ii) the coverage percentage will be equal to 100%, whenever the numerator value is equal to or greater than the denominator value.

The database produced within the scope of this paper is stored in Synapse – in Portuguese (original language) to favor its use among Brazilian health professionals and researchers and in English for ease of use by the international community. The database has 184,801 observations and 21 variables.

In addition to geographic coverage, year, semester, name, and location code, there are six variables with the absolute numbers of children and families with a health profile, monitored and with up to date conditionalities, four variables on pregnant women, and six coverage variables.^3^ The numbers of families and children with a health profile comprised the denominators of the percentages of coverage of monitoring of the PBF’s health conditionalities. The percentages of coverage of compliance with health conditionalities, in turn, have as denominators the number of families and children benefiting from the PBF monitored by the SUS and, in the case of pregnant women, the total number of pregnant women located.

## Limitations

- Possibility of analyzing only data aggregated at the geographic level.
- In 2019/1, the CNS field (National Health Registry number) was inserted in the BFA System of the e-Gestor AB platform, favoring the migration of data from the e-SUS AB - which presumably led to an increase in the number of families beneficiaries in the BFA System.
- The estimation of the number of pregnant women in the municipalities was not checked.
- The data does not provide reasons for not following up on families with a health profile and non-compliance with the PBF’s health conditionalities.

## Data Availability

The dataset generated during the current study and additional documentation is freely and openly available on the Synapse repository at https://doi.org/10.7303/syn25884313 [8]. Please see Table 1 for details.

## Abbreviations

PBF: “Bolsa Família” Program
BFA: “Bolsa Família” Program in Health
SUS: Unified Health System

## Footnotes

1 Until 2018, data from the PBF Health System (BFA) are available at: http://bolsafamilia.datasus.gov.br/.

2 As of 2018, the BFA System data are available at: https://bfa.saude.gov.br/.

3 More detailed information can be found at https://www.synapse.org/Synapse:syn25885153.
